# Magnetic resonance imaging findings in painful hemiplegic shoulder patients with or without subluxation: A retrospective cohort study

**DOI:** 10.3389/fneur.2022.1032676

**Published:** 2022-11-14

**Authors:** Hui-Min Xie, Xiao-Tan Zhang, Lin Xu, Ning Wang, Rui Wang, Zi-Shan Jia, Li-Ning Zhang

**Affiliations:** ^1^Department of Rehabilitation Medicine, The First Medical Centre, Chinese PLA General Hospital, Beijng, China; ^2^Department of Radiology, The First Medical Centre, Chinese PLA General Hospital, Beijng, China

**Keywords:** magnetic resonance imaging, shoulder pain, hemiplegic shoulder, glenohumeral subluxation, hemiplegic shoulder pain

## Abstract

The relationship between hemiplegic shoulder pain (HSP) and subluxation is unclear. This study aimed to determine the differences of magnetic resonance imaging (MRI) findings in HSP patients with or without subluxation after stroke, and to analyze the etiology of shoulder pain. This retrospective study included 53 patients with HSP after stroke from September 2013 to February 2020. Patients underwent MRI of the shoulder because of shoulder pain. Clinical characteristics, including age, sex, stroke duration, body mass index, stroke type, visual analog scale score, Brunnstrom stage, and MRI arthrography findings of the affected shoulder, were recorded. Patients were classified into the glenohumeral subluxation (GHS) group (*n* = 27) or non-glenohumeral subluxation (nGHS) group (*n* = 26). We found that patients with HSP may be prone to bursa effusion, rotator cuff injury, ligament injury, and cartilage injury, even though there was no significant difference between the GHS and nGHS groups. MRI revealed 14 cases of long bicipital tendon-glenoid labrum injury (51.8%) in the GHS group and 6 cases (23.1%) in the nGHS group (*p* = 0.030). We also found 10 cases (37%) of glenoid labrum injury in the GHS group and 2 cases (7.7%) in the nGHS group (*p* = 0.026). Eight cases (29.6%) and 1 case (3.8%) of bone marrow edema were found in the GHS and nGHS groups, respectively (*p* = 0.033). Compared with painful hemiplegic shoulder patients without subluxation, patients with subluxation may be more susceptible to some injuries, such as long bicipital tendon-glenoid labrum injury, glenoid labrum injury, and bone marrow edema. During rehabilitation, physicians need to pay attention to these injuries.

## Introduction

Stroke often causes disability among elderly people. Hemiplegic shoulder pain (HSP) is one of the most common complications in patients after a stroke. The incidence of HSP is approximately 17–72% (1). HSP, which is related to depression and a poor quality of life, negatively affects functional recovery of the upper extremity and activities of daily living (ADLs) (2). The pathogenesis of HSP includes shoulder subluxation, adhesive capsulitis, bursitis, shoulder-hand syndrome, among others (3). However, the exact etiology of HSP remains unknown and many complicated factors are involved. Glenohumeral subluxation (GHS) may be considered a potential cause of shoulder pain development (4).

The prevalence of GHS was reported to be 15–81% (2). There is speculation that the peri-articular tissue of the shoulder may be overstretched because of malalignment of the joint. The capsule and ligaments contain high concentrations of pain receptors, which cause shoulder pain (5).

Factors contributing to joint malalignment include rotator cuff weakness, loose ligaments and capsule, and impingement between the humeral head and shoulder suture. GHS commonly occurs in the flaccid stage, which is characterized by areflexia and atonia (6). However, the relationship between HSP and subluxation is unclear. Magnetic resonance imaging (MRI), which is widely used in diagnosing and determining the pathologies of HSP, has been proven to be more advantageous than other imaging techniques because it can clearly show the details of the soft tissue (7).

Thus, the present investigation aimed to determine the differences between MRI findings in HSP patients with subluxation and non-subluxation after stroke, and to analyze the etiology of shoulder pain.

## Materials and methods

### Patient selection

**Figure 1:**
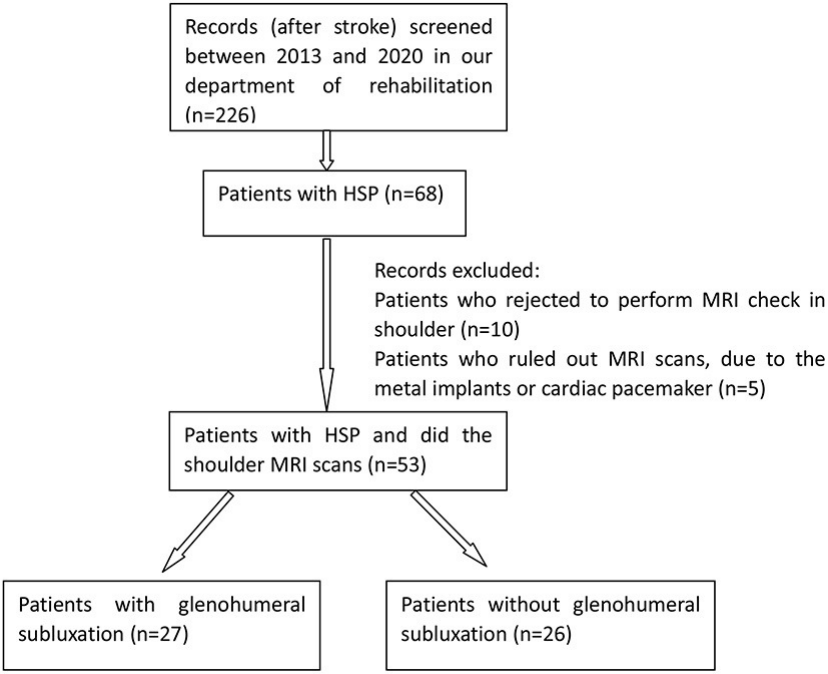
Flow chart and retention of patients.

This was a retrospective cohort study conducted at the rehabilitation center of the First Medical Centre, Chinese PLA General Hospital in Beijing. The data of 53 post-stroke patients with HSP were collected for this study from September 2013 to February 2020 (Figure 1). Inclusion criteria were first-time stroke resulting in HSP and no history of shoulder disorder before stroke onset. Exclusion criteria were history of shoulder trauma and surgery, and severe cognitive impairment. Patients with GHS were categorized into the GHS group, and those without GHS were allocated into the non-glenohumeral subluxation (nGHS) group.

### Ethical approval

This study was approved by the Ethics Committee of the Chinese PLA General Hospital (No. S2019-230-01). As an anonymous retrospective study, the need for obtaining informed consent from patients was waived.

### Definitions and data collection

The diagnosis of stoke was confirmed based on the patients' history, clinical symptoms, physical examinations, and computed tomography/MRI findings. HSP was diagnosed on the basis of two criteria: visual analog scale (VAS) score (≥4) and limited passive range of motion of the affected shoulder (reduced shoulder abduction and external rotation ≥25%) (8).

GHS was diagnosed based on the clinical palpation method, which has been shown to be a reliable screening measure with good inter- and intra-rater reliabilities (9, 10). The patient was seated with their arm relaxed beside their body. The distance between the acromion and humeral head was measured. If the distance was longer than a fingerbreadth, it indicated the presence of subluxation (6). The diagnosis of GHS was made by two examiners, a rehabilitation physician and a physiotherapist, both of whom had > 5 years of experience with stroke patients in the rehabilitation department.

Shoulder MRI was performed once shoulder pain occurred; 3.0 Tesla Skyra MRI (Siemens) was used. Patients were positioned supine with their upper limb in a neutral position. An identical MRI protocol of the hemiplegic shoulder was used for all patients, as follows: T1WI-SAG and T1WI-COR: TE/TR 22/600ms, PDWI-SAG: TE/TR 37/3800ms, PDWI-COR: TE/TR 42/3100ms, PDWI-TRA: TE/TR 73/3780ms. The field of view was set to 18 cm, and the image sequences were obtained with a matrix acquisition range of 320 × 256. The slice thickness was 4 mm. The images were read individually by two experienced radiologists.

Age, sex, body weight, height, body mass index (BMI), hemiplegic side, stroke type (ischemic or hemorrhagic), and stroke duration were recorded. The level of motor function was assessed by the Brunnstrom recovery stages of the upper extremity (9), which were defined as follows: I, flaccid stage without any voluntarily muscle movement; II, muscle contraction with weak flexor and/or extensor synergies; III, voluntary movement of the upper limbs without selective activation; IV, selective activation coming; V, more predominant selective activation; VI, proper coordination of isolated movements ignoring speed. The pain of the affected shoulder was evaluated by the VAS. Patients scored the intensity of their shoulder pain in person on a scale from 0 to 10 (10). A VAS score of 0 was defined as no pain, and 10 as the worst pain. The Barthel Index was used to assess patients' ADLs. The Barthel Index is considered to be the best ADL measurement scale. Barthel Index scores are based on the completion status of some tasks, such as bathing, feeding, toileting, stair climbing, dressing, personal hygiene, bowel control, bladder control, ambulation, and chair/bed transfers (11). BMI was determined as weight (kg) divided by height (m)^2^.

### Data analysis

Statistical analysis was performed using SPSS statistical software (version 23; IBM Corp.). The independent samples t-test was used to compare the differences in age, stroke duration, BMI, VAS, and Barthel scores between the groups, while the Pearson's chi-squared test test was used to compare the differences in sex, stroke type, hemiplegic side, and paresthesia between the groups. Normality of the data distribution was checked using the Kolmogorov–Smirnov test. Additionally, the Wilcoxon rank-sum test was used to compare differences in the Brunnstrom recovery stage between the groups. Further, the Pearson's chi-squared test, and Fisher's exact test were utilized to compare differences in MRI findings between the groups. In cases of cells <5 and >0, the chi-square test was used. In case of cells equal to 0 or total numbers <40, Fisher's exact test was used. Statistical significance was defined as *p* <0.05.

## Results

### Clinical characteristics of the sample

Fifty-three patients (39 men and 14 women; age range, 22–80 years) were included in our study. The GHS group comprised 27 patients, and the nGHS group comprised 26 patients. Mean stroke durations were 89.5 days in the GHS group and 102.15 days in the nGHS group. The numbers of patients with paresthesia in the GHS and nGHS groups were 16 and 10, respectively. There was no significant difference between the groups in age, sex, BMI, stroke duration, hemiplegic side, stroke type, and paresthesia (*p* > 0.05). In the GHS group, the mean Barthel score was 42.4, but in the nGHS group, the mean score was 59.6 (*p* = 0.616). Mean VAS scores were 6.3 and 5.2 in the GHS and nGHS groups, respectively (*p* = 0.375). No significant differences were found in the Barthel and VAS scores between the groups. The ratios of the Brunnstrom stages I/II/III/IV/V/VI in the groups were 2/14/10/0/1/0 and 0/6/10/3/7/0, respectively. There was a significant difference in the Brunnstrom stage between the groups (*p* = 0.002). Demographic characteristics of the two groups are shown in Table 1.

**Table 1:** Clinical features of the patients.

	**GHS group**	**nGHS group**	***P*** **-value**
	**(*n* = 27)**	**(*n* = 26)**	
Age, y, mean (SD)	59.7 (11.4)	56.27 (13.1)	0.082
Gender, female/male, *n*	19/8	20/6	0.589
Stroke duration, *d*, mean (SD)	89.5 (62.8)	102.15 (154)	0.296
BMI, cm/kg^2^, mean (SD)	23.7 (3.4)	24.8 (4.1)	0.218
**Stroke type (** ***n*** **)**			0.407
Ischemic	18	20	
Hemorrhage	9	6	
**Hemiplegic side (** ***n*** **)**			0.449
Left	16	18	
Right	11	8	
Paresthesia, *n* (%)	16 (0.59)	10 (0.37)	0.13
Barthel Index, mean (SD)	42.4 (23)	59.6 (22.2)	0.616
VAS, mean (SD)	6.3 (1.7)	5.2 (3.2)	0.375
**Brunnstrom stage**, ***n*** **(%)**			0.002^*^
I	2	0	
II	14	6	
III	10	10	
IV	0	3	
V	1	7	
VI	0	0	

BMI, body mass index; VAS, visual analog scale; y, year; d, day.

* Denotes statistical significance.

### MRI findings

MRI findings included ligament injury, rotator cuff injury, long head of the biceps tendon injury, bursa effusion, cartilage injury, synovitis, bone morrow edema (BME), long bicipital tendon-glenoid labrum injury, and glenoid labrum injury. No significant differences were found in rotator cuff injury, bursa effusion, cartilage injury, ligament injury, and synovitis between the groups.

**Figure 2:**
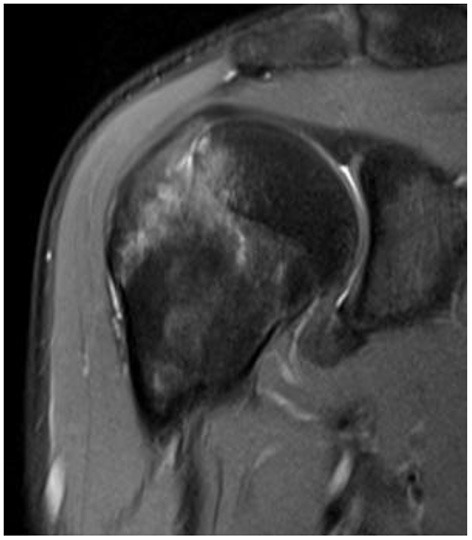
Fat suppressed T2 images. The bone marrow edema is shown.

**Figure 3:**
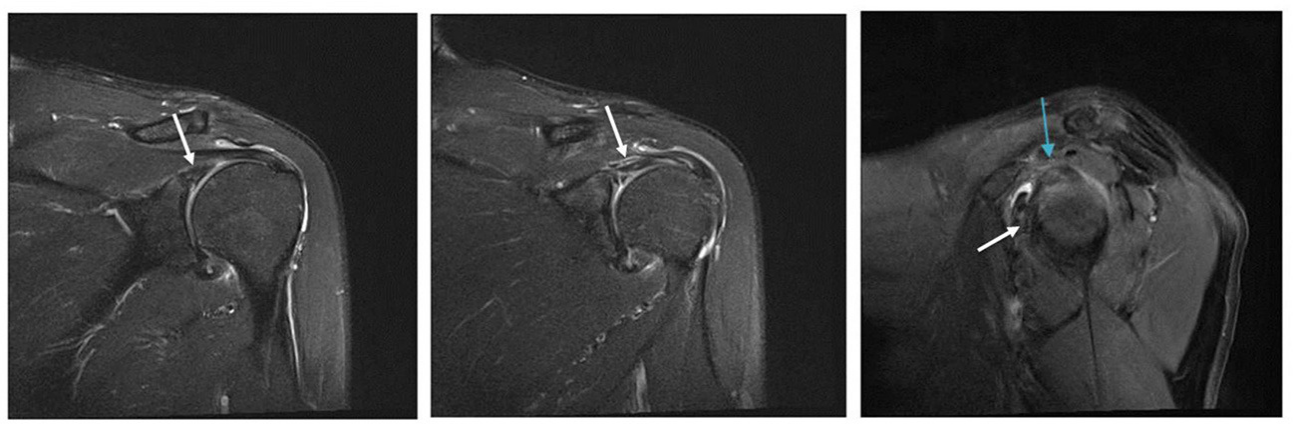
Fat suppressed T2 images. The long bicipital tendon-glenoid labrum injury is shown by the white arrow. The sup-glenoid labrum injury is shown by the blue arrow.

**Figure 4:**
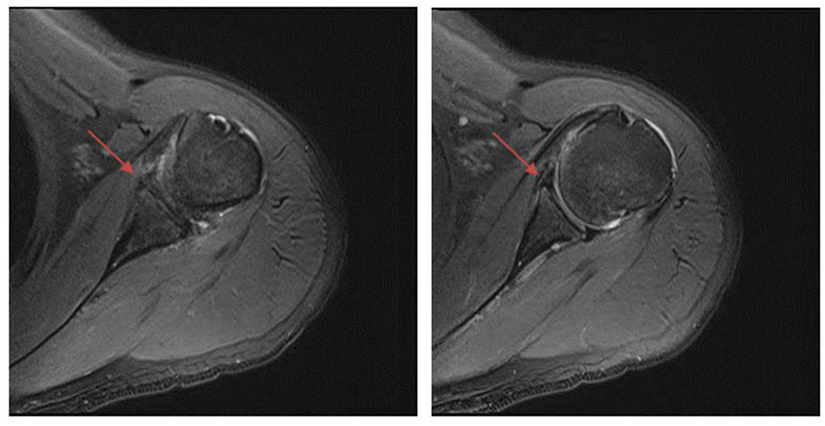
Fat suppressed T2 images. The anterior labrum injury is shown by the red arrow.

In the GHS group, 8/27 patients (29.6%) had a positive MRI finding of BME, but in the nGHS group, only 1/26 patients (3.8%) had such finding (*p* = 0.033). We also found that 14/27 patients (51.8%) had a long bicipital tendon-glenoid labrum injury in the GHS group, whereas 6/26 patients (23.1%) had such injury in the nGHS group (*p* = 0.030). Additionally, 10/27 patients (37.0%) in the GHS group and only 2/26 patients (7.7%) in the nGHS group had a glenoid labrum injury (*p* = 0.026). In the GHS group, 4 patients had extensive injury of the glenoid labrum, and 6 had partial injury (anterior and superior portion 1; posterior and superior portion 1; anterior and inferior portion 1; anterior portion 1; posterior portion 1). In the nGHS group, only 2 patients had partial injury of the glenoid labrum (anterior and superior portion 1; anterior portion 1). See Table 2 for details and Figures 2–4 for examples.

**Table 2:** Comparison of MRI findings between GHS group and nGHS group.

	**GHS group**	**nGHS group**	***P*** **-value**
	**(*n* = 27)**	**(*n* = 26)**	
**Bursa effusion**	26 (96.3)	26 (100)	NA
Subacromial-subdeltoid bursa	19 (70.4)	19 (73.1)	0.827
Subcoracoid bursa	25 (92.6)	21 (80.8)	0.387
Subscapular bursa-cavity	22 (81.5)	24 (92.3)	0.448
**Rotator cuff injury**	26 (96.3)	24 (92.3)	0.973
Supraspinatus	25 (92.6)	20 (76.9)	0.227
Infraspinatus	6 (22.2)	2 (7.7)	0.274
Subscapularis	14 (51.9)	14 (53.8)	0.884
Teres Minor	3 (11.1)	0	0.236
Ligament injury	5 (18.5)	4 (15.4)	0.906
Effusion or tendinosis of long head of biceps tendon	20 (74.1)	21 (80.8)	0.56
Synovitis	7 (25.9)	5 (19.2)	0.56
Cartilage injury	3 (11.1)	0	0.236
Bone morrow edema	8 (29.6)	1 (3.8)	0.033^*^
Long bicipital tendon- glenoid labrum injury	14 (51.8)	6 (23.1)	0.030^*^
**Glenoid labrum injury**	10 (37.0)	2 (7.7)	0.026^*^
Ext	4 (14.8)	0	
Ant, Sup	3 (11.1)	1 (3.8)	
Post, Sup	1 (3.7)	0	
Ant, Inf	1 (3.7)	0	
Ant	1 (3.7)	1 (3.8)	
Post	1 (3.7)	0	

GHS, Glenohumeral subluxation; Nghs, non-glenohumeral subluxation; Ant, anterior; Post, posterior; Sup, superior; Inf, inferior; Ext, Extensive.

* Denotes statistical significance.

## Discussion

In this study, MRI of the affected shoulder was performed in patients with or without subluxation. Shoulder subluxation occurs mostly during the first 3 weeks in patients with HSP after a stroke (12). The incidence rate was reported to range from 32 to 81% (13), and the rate found in our study (51%) falls within this range.

The MRI scans demonstrated rotator cuff injury in 94% of the shoulders of patients in our study. However, in a study conducted by Dogun et al., 63.2% of patients (*n* = 68) with HSP were found to have a rotator cuff injury (7). The difference between their study and ours may be attributed to the different stroke onset durations: Their mean duration was 49 days, whereas ours was 85. Moreover, we found that the rotator cuff injury rate was high regardless of subluxation. In detail, supraspinatus injury rates were 92.6% in the GHS group and 76.9% in the nGHS group. Moreover, about half of patients had subscapularis injury. Contrastingly, the incidence of infraspinatus and teres minor injury was low. The main function of the supraspinatus is to abduct the shoulder, and it is an important posterior stabilizing structure of the shoulder. According to several studies (14, 15) supraspinatus injury is mainly caused by subacromial impingement. When the shoulder is frequently abducted and lifted upward, the supraspinatus tendon becomes easily impacted by the coracoacromial arch, resulting in edema, hyperemia, degeneration, and even tearing. Zhu et al. showed that when the shoulder was abducted 60°, it bore the greatest stress; additionally, it was beginning to rub between the supraspinatus tendon and the acromion and was thus most prone to injury and pain (16).

The functions of the subscapularis are to internally rotate the shoulder and dynamically stabilize the humeral head. Moreover, the subscapularis is an antagonist to the superior pull of the deltoid and it assists in abduction and adduction of the shoulder (17). External rotation and abduction can cause excessive strain injury of the subscapularis, which results in pain in the front of the shoulder (18). In addition, a frequent overhead throwing motion can cause coracoid impingement, which is closely associated with subscapularis injury. Therefore, rotator cuff injury, especially supraspinatus and subscapularis injury, is one of the causes of HSP. It is important for rehabilitation teams to not schedule exercises that involve moving the upper limbs above 60° with abduction action in patients with HSP. Furthermore, if the patient has a possible subscapular injury, external rotation of the shoulder should be avoided as much as possible.

Supraspinatus injury was recently hypothesized to result in compensation *via* greater force generation through the subscapularis, which could potentially hasten degeneration of the subscapularis (19). Accordingly, we could infer that there may be a correlation between supraspinatus and subscapularis injuries in patients with HSP, but this needs to be confirmed by a study with a large sample size.

Our MRI scans also demonstrated subacromial-subdeltoid bursa effusion in 71.74% of shoulders in patients with HSP, which is consistent with previous reports (53–80.9%) (7, 20, 21). Besides, we found a high subcoracoid bursa effusion and subscapular bursa-cavity effusion rate, even though this was not statistically significant between the groups. It has been suggested that subcoracoid bursa effusion and subscapular bursa-cavity effusion, except subacromial-subdeltoid bursa effusion, may cause HSP.

In a study of 42 fresh cadaveric shoulders, it was found that the function of the subscapularis and subcoracoid bursae is to manage friction of the superficial fibers against the scapular neck, humeral head, and coracoid process (22). Thus, it remains to be explored whether friction of these bone structures is increased after biomechanical changes in hemiplegic patients, resulting in fluid accumulation in the bursa.

Thanks to MRI, BME has been detected in the humeral head of numerous patients with HSP: BME was mostly reported in the femoral head and knee (23, 24). In recent studies, instances of BME were observed in the foot, ankle, wrist, or other bones (25, 26). There are two perspectives about the mechanism of BME: (1) secondary BME, secondary to infection, trauma, or arthritis, is caused by an external force acting on the cancellous bone, resulting in a microfracture of the trabecula bone that increases permeability and rupture of the local capillary, adding to the exosmosis of cell fluid and vascular perfusion; and (2) physiological BME is caused by a long-term external force or change of the normal load of the bone, resulting in bone marrow hyperemia and excessive perfusion of the capillaries. In our study, BME occurred more in patients with subluxation which may be due to (1) rotator cuff weakness and change in gravity that caused secondary BME and (2) excessive passive movement or incorrect posture that caused physiological BME in flaccid paralysis period. Because of its self-limiting course (24), BME could improve after the paralysis period. Thus, the potential evolution and mechanism of BME in the shoulder requires further study.

Long bicipital tendon-glenoid labrum injury occurred more commonly in the GHS group. It is caused by repeated contraction of the long head of the bicipital tendon or trauma to the humeral head with repeated external rotation and abduction movements (27). Thus, when rehabilitating patients with HSP, especially those with subluxation, physicians should avoid such movements.

We usually divide the labrum into eight directions: anterior, posterior, superior, inferior, anterior superior, anterior inferior, posterior superior, and posterior inferior. We also briefly differentiated the exact location of the simple labrum injury observed herein. MRI findings showed that simple labrum injury was more common in the GHS group than in the nGHS group, and it included extensive anterior superior, posterior superior, anterior inferior, anterior, and posterior injury. Our finding that extensive injury in the anterior superior was the most common injury type differs from findings of shoulder imaging in cases of sports injury (27). This discrepancy implies that the biological stress produced by passive motion is different from that of active motion.

The limitations of this study include the small sample size, absence of MRI images of contralateral healthy shoulders, and retrospective design. A larger scale, controlled clinical trial with a longer-term, longitudinal follow-up is warranted.

## Conclusion

On the basis of our MRI findings, we found a high frequency of bursa effusion, rotator cuff injury, and long head of the biceps tendon injury in patients with HSP. Compared to patients with HSP in the non-subluxation group, we found that patients with HSP in the subluxation group were more prone to BME, long bicipital tendon-glenoid labrum injury, and glenoid labrum injury. Thus, in patients with HSP, we recommend that physicians avoid moving the upper limbs above 60° with abduction action. If patients have subluxation, physicians and therapists should plan to reduce the external rotation and abduction movements to prevent rotator cuff injury and long bicipital tendon-glenoid labrum injury during rehabilitation. Patients with subluxation should be moved gently if they require passive actions. Moreover, they should be educated about protecting their shoulders during ADLs. Further studies with a larger sample size are needed to confirm our findings.

## Data availability statement

The original contributions presented in the study are included in the article/supplementary material, further inquiries can be directed to the corresponding authors.

## Ethics statement

The studies involving human participants were reviewed and approved by No. S2019 230 01. Written informed consent for participation was not required for this study in accordance with the national legislation and the institutional requirements. Written informed consent was obtained from the individual(s) for the publication of any potentially identifiable images or data included in this article.

## Author contributions

Z-SJ, X-TZ, and L-NZ designed the experiment. H-MX and LX read and checked the MRI images. NW and RW assessed the clinical information of the patients. H-MX wrote the draft of the manuscript. All authors discussed the results, commented on the manuscript, and approved the submitted version.

## Conflict of interest

The authors declare that the research was conducted in the absence of any commercial or financial relationships that could be construed as a potential conflict of interest.

## Publisher's note

All claims expressed in this article are solely those of the authors and do not necessarily represent those of their affiliated organizations, or those of the publisher, the editors and the reviewers. Any product that may be evaluated in this article, or claim that may be made by its manufacturer, is not guaranteed or endorsed by the publisher.

## Abbreviations

HSP: hemiplegic shoulder pain
GHS: glenohumeral subluxation
MRI: magnetic resonance imaging
VAS: visual analog scale
BMI: body mass index
ADL: activity of daily living
TR: repetition time
TE: echo time
BME: bone marrow edema.
